# Sex-specific associations between prenatal maternal mental health and child behavior problems at age 7: A (multi-center) longitudinal study of socially disadvantaged mother-child dyads

**DOI:** 10.1007/s00787-026-02974-z

**Published:** 2026-02-10

**Authors:** Meret Lakeberg, Christian Bachmann, Falk Hoffmann, Tanja Jungmann, Malte Sandner, Hajo Zeeb, Sören Kliem, Tilman Brand

**Affiliations:** 1 Department of Health Services Research, Carl von Ossietzky University, Oldenburg, Germany; 2 Department of Child and Adolescent Psychiatry, University of Ulm, Ulm, Germany; 3 Department of Special Needs Education and Rehabilitation, Carl von Ossietzky University Oldenburg, Oldenburg, Germany; 4 Department of Business Administration, Nuremberg Institute of Technology, Nuremberg, Germany; 5 Department of Prevention and Evaluation, Leibniz Institute for Prevention Research and Epidemiology Bremen, Bremen, Germany; 6 Department of Social Work, University of Applied Sciences Jena, Jena, Germany

**Keywords:** Child behavior, Child behavior problems, Maternal mental health, DASS-21, CBCL

## Abstract

Mental health issues are highly prevalent in socially disadvantaged populations, impacting both mothers and children. Maternal mental health plays a crucial role in child development and well-being. This study aimed to assess the association between maternal symptoms of depression, anxiety and stress during pregnancy and child behavioral problems at age 7 in a socially disadvantaged population. Data from the German longitudinal Pro Kind study were analyzed using the Depression-Anxiety-Stress Scale (DASS-21) to assess maternal distress in pregnancy and the Child Behavior Checklist (CBCL 6-18R) to measure internalizing and externalizing problems at age 7. Logistic regression models were applied, adjusting for potential confounders, including sex-stratified analyses. The study sample included 508 socially disadvantaged mother-child dyads, with a high prevalence of maternal mental distress and a substantial proportion of children exhibiting behavioral problems. In female children, moderate to severe maternal depression, anxiety, and stress during pregnancy increased the likelihood of internalizing problems as well as the total behavior problems at age 7. In male children, only mild, but not moderate or severe symptoms of maternal stress were associated with an increased likelihood of an elevated total problem score at age 7. The results of this study highlight the importance of maternal mental health in pregnancy as a predictor for child mental health, and also point to potential sex/gender differences in susceptibility to child problem behavior associated with maternal distress. Our findings underscore the need for targeted prenatal mental health interventions, especially within socially disadvantaged populations.

**Supplementary Information:**

The online version contains supplementary material available at 10.1007/s00787-026-02974-z.

## Introduction

Mental health conditions are a significant public health concern worldwide. In Germany, they rank as the second-largest contributor to direct healthcare costs, surpassed only by cardiovascular diseases (Federal Statistical Office [ 16]. A representative German study from 2014 reported a 12-month prevalence of mental disorders of 27.7%. This prevalence is even higher among individuals with low socioeconomic status, reaching 37.9% [22].

Mental health problems not only affect the individual directly but can also have far-reaching consequences for future generations. Extensive research has demonstrated that maternal psychological distress—including depression, anxiety, and stress—significantly increases the risk of behavioral problems in children later in life [3, 5, 34, 36, 42]. Empirical evidence further highlights the critical role of prenatal maternal mental health. A 2020 meta-analysis found a strong association between maternal prenatal depression and anxiety and adverse developmental outcomes in children. Poor maternal mental health during pregnancy was linked to impairments in children’s social-emotional, language, and motor development [36]. In addition to maternal mental health during pregnancy, several other risk factors have been identified as contributing to child behavioral problems. These include smoking or substance use, inadequate intake of essential nutrients, pregnancy complications such as preeclampsia or preterm birth, exposure to environmental toxins, genetic predispositions, and epigenetic changes [2, 8, 14, 18, 20, 40].

Despite the substantial body of research on the impact of prenatal maternal psychological distress on child health, several key questions remain unanswered [30, 36]. While many studies have focused on short-term outcomes, particularly in infancy and early childhood, fewer have examined the effects on behavioral problems in middle childhood. The present study aims to address this gap by investigating the long-term impact of maternal psychological distress during pregnancy on children at the age of seven. Evidence on this association is particularly scarce in socially disadvantaged populations, where maternal distress tends to be more prevalent.

Another important yet understudied area is the potential role of sex differences in this relationship. To date, few studies have applied appropriate analytical methods to investigate sex-specific effects, and often, these differences are not examined at all [36, 42]. However, some research suggests sex-specific susceptibility to maternal distress during pregnancy. For example, a cohort study found that prenatal anxiety was associated with externalizing problems in preschool-aged boys, while girls were more likely to develop internalizing problems [44]. Similarly, a longitudinal study reported that maternal emotional complaints—including depressive and anxious symptoms—during the first trimester were associated with internalizing problems in boys. In contrast, girls appeared more vulnerable to maternal distress in the third trimester, exhibiting both internalizing and externalizing problems in early childhood [11]. Another study including only mothers with low income found that prenatal depressive symptoms were linked to increased toddler behavior problems, and that maternal sensitivity moderated these effects in a sex-specific manner: boys were more negatively affected by low maternal sensitivity, whereas girls benefitted more strongly from high maternal sensitivity [15].

Additional evidence from longitudinal studies focusing on the postnatal wellbeing of the mother further support the notion of sex-specific vulnerability. [45] showed that mothers with high levels of depressive symptoms displayed less adaptive interactional repair with their infants, with particularly negative consequences for boys, who exhibited reduced recovery from stress. [28] demonstrated that both prenatal and postnatal maternal depressive symptoms predicted poorer adolescent well-being, with girls showing stronger associations with internalizing problems, while boys were more prone to externalizing problems. In light of the mentioned research gaps, the present study aims to investigate the relationship between maternal psychological distress during pregnancy and behavioral problems in children at the age of seven, with particular attention to sex-specific differences.

## Methods

### Data

Data for this analysis were derived from the German longitudinal study *Pro Kind*. The study initially conducted to evaluate the effects of a home visitation program delivered by social workers and family midwives over two years on maternal competencies, family environment, and child development in 15 municipalities across three German federal states. The women were included in the baseline measurement (T0) in the second trimester of pregnancy (12th −28th week) (enrolment period 2006–2009). Follow-ups were conducted at the 36th week of pregnancy (T1), and at 6 (T2), 12 (T3), and 24 (T4) months postpartum, as well as 7 years after birth (T5) [26]. For the present study, mainly data from the baseline (t0) and the 7-year follow-up (T5) were analyzed plus some additional data from T1 and T2. For this analysis, we combined the control and the intervention group, because the intervention was not the primary focus of this analysis.

Inclusion criteria for the women in the study were no previous live birth, being in the second trimester of pregnancy, low income, plus at least one additional psychosocial risk factor such as an underage pregnancy or experiences of abuse. Participants were recruited through various stakeholders, such as gynecologists, youth welfare offices or employment agencies.

The study design and procedures were approved by the Ethics Committee of the German Psychological Society as well as the Ethics Committee of Leipzig University.

## Measures

Data collection involved computer-assisted telephone interviews and face-to-face interviews with participating women conducted by trained interviewers [25, 38].

The Depression-Anxiety-Stress-Scale (DASS-21) was employed to assess the self-reported mental health status of the mothers at baseline. The 21-item questionnaire evaluates symptoms experienced over the previous week and is divided into three subscales: *depression*,* anxiety*, and *stress*, each comprising seven items. Response options range from 0 (“did not apply to me at all”) to 3 (“applied to me very much or most of the time”). The *depression scale* captured symptoms such as dysphoric mood, lack of drive, and hopelessness (normal: 0–4, mild: 5–6, moderate: 7–10, severe: 11–13, extremely severe: >14) while the *anxiety scale* measured symptoms of fear, panic, and physiological arousal (normal: 0–3, mild: 4, moderate: 5–7, severe: 8–9, extremely severe: >10). The *stress scale* focused on irritability, tension, and physical discomfort (normal: 0–7, mild: 8–9, moderate: 10–12, severe: 13–16, extremely severe: >17). Responses can be analyzed by numerical scores or grouped into ordinal categories [32].

The CBCL 6-18R was administered at T5 to assess behavioral problems, somatic symptoms, and social skills among the children within the study population. Widely regarded as a reliable tool, the CBCL contains 113 items that were rated by the mother based on the child’s behavior over the past six months. Each item was scored as 0 (“not true”), 1 (“partly or sometimes true”), or 2 (“very or mostly true”). The items were grouped into three major problem scales: *internalizing problems*,* externalizing problems*, and the *total problems scale*. *Internalizing problems* include the subcategories of anxious/depressive behavior, withdrawn/depressive behavior, and somatic complaints. *Externalizing problems* encompass rule-breaking and aggressive behaviors. The *total problems scale* combines internalizing and externalizing problems with additional subscales, including social problems, thought problems, sleep issues, repetitive behaviors, and attention problems. CBCL results are interpreted based on T-scores normed by the child’s sex and age. A T-score above 60 falls within the clinical range for behavior problems [1, 37].

In addition, sociodemographic data were collected, including the mother’s and child’s age, child’s sex, mother’s nationality, and educational attainment. Further data were collected on risk factors, including teenage pregnancy, single parenthood, self-reported alcohol consumption and smoking during pregnancy, as well as experiences of neglect and/or abuse during the mother’s childhood. Information on preeclampsia during pregnancy and the occurrence of low birth weight (< 2500 g) was collected from maternity records at T1 and T2. Additionally, the presence of siblings in the household at T5 was recorded.

For study participants to be included in this analysis, results of the CBCL questionnaire and information on the child’s sex had to be available.

### Statistical analysis

Multiple logistic regression analyses were used to examine the association between maternal mental health status (DASS-21) regarding depression, anxiety, and stress in pregnancy and child behavioral problems (CBCL 6-18R) at the age of seven. The probability of child behavioral problems was modeled in relation to clinical cut-off values for both the child’s behavior and the mother’s mental well-being. Odds ratios (OR) and 95% confidence intervals (95%-CI) were calculated. Sex-stratified logistic regression analyses were conducted to explore differences in the associations across child sexes.

Due to missing data, multiple imputations were conducted following the fully conditional specification (FCS) method, a statistically valid method for creating imputations in a dataset that includes categorical and continuous variables [29]. The number of imputations was determined based on the percentage of missing values, resulting in 47 imputations. All variables were used for imputation of missing values and used as covariates in the regression analyses. The regressions were run both with the imputed dataset and the complete cases for comparison.

All analyses were conducted using the Statistical Analysis System (SAS 9.4).

## Results

### Participants characteristics

Of the 755 women enrolled at baseline, the analytical sample of this study consisted of 508 mothers with their children, by including only mother-child dyads who completed the CBCL questionnaire (Table 1). The mean age of the mothers at baseline was 21.5 years (SD 4.3; range 14.2–40.3 years). The children were, on average, 7.5 years old (SD 0.7) at the follow-up. The sex distribution of the children was nearly balanced with 266 female and 240 male children, while the information on sex of two children was missing. 16.5% of the mothers reported a non-German nationality. Most mothers had no school leaving certificate at baseline (92.4%).

**Table 1:** Descriptive characteristics for the total sample, stratified by child’s sex (*n* = 508)

Demographic characteristics		Mean (SD)		
		female	male	total
Mothers age at baseline (*n* = 508)		21.5 (4.3)	22.0 (4.6)	21.7 (4.4)
Child’s age at follow-up (*n* = 506)		7.5 (0.7)	7.5 (0.7)	7.5 (0.7)
		**n (%)**		
Non-German nationality (*n* = 506)		45 (17.0)	38 (15.9)	83 (16.5)
Mothers’ school education at baseline (*n* = 497)	No school leaving certificate	241 (92.7)	216 (91.9)	459 (92.4)
	Certificate of Primary or Secondary Education	12 (4.6)	12 (5.1)	24 (4.8)
	University entrance qualification	7 (2.7)	7 (3.0)	14 (2.8)
Teenage pregnancy (*n* = 508)		48 (18.0)	35 (14.6)	83 (16.4)
Alcohol consumption during pregnancy (t0) (*n* = 505)		20 (7.6)	23 (9.6)	43 (8.5)
Occasional or daily smoking during pregnancy (t0) (*n* = 506)		131 (49.4)	111 (46.4)	242 (48.0)
No partnership (t0) (*n* = 508)		85 (32.0)	68 (28.3)	153 (30.2)
Maternal experiences of abuse/neglect in childhood (*n* = 490)		98 (38.1)	79 (34.2)	177 (36.1)
Presence of siblings (t5) (*n* = 472)		118 (47.2)	119 (53.6)	237 (50.2)
Preeclampsia (*n* = 374)		25 (12.3)	15 (8.7)	40 (10.6)
Low birth weight (*n* = 379)		13 (6.4)	5 (2.8)	18 (4.8)
Depression (DASS-21) (*n* = 501)	Normal	156 (59.3)	128 (54.2)	286 (57.1)
	Mild	31 (11.8)	35 (14.8)	66 (13.2)
	Moderate	46 (17.5)	47 (19.9)	93 (18.6)
	(Extremely) severe	30 (11.4)	26 (11.0)	56 (11.2)
Anxiety (DASS-21) (*n* = 499)	Normal	138 (52.7)	119 (50.6)	258 (51.7)
	Mild	38 (14.5)	25 (10.6)	63 (12.6)
	Moderate	51 (19.5)	54 (23.0)	106 (21.2)
	(Extremely) severe	35 (13.4)	37 (15.7)	72 (14.4)
Stress (DASS-21) (*n* = 500)	Normal	77 (29.4)	73 (30.9)	151 (30.2)
	Mild	42 (16.0)	26 (11.0)	68 (13.6)
	Moderate	67 (25.6)	71 (30.1)	138 (27.6)
	(Extremely) severe	76 (29.0)	66 (28.0)	143 (28.6)
Internal problems (CBCL) (*n* = 400)		92 (42.0)	84 (46.4)	176 (44.0)
External problems (CBCL) (*n* = 482)		140 (54.9)	110 (48.5)	250 (51.9)
Total problems (CBCL) (*n* = 371)		120 (59.1)	100 (59.5)	220 (59.3)

A total of 16.4% of the mothers were younger than 18 years at the time of pregnancy. Alcohol consumption during pregnancy was reported by 8.5% of mothers, while 48.0% of mothers reported occasional or daily smoking. A total of 30.2% of mothers reported having no partnership at baseline. In terms of maternal mental health during pregnancy, 18.6% of the mothers showed moderate and 11.2% (extremely) severe depressive symptoms. 21.2% had moderate symptoms of anxiety, while 14.4% showed a severe outcome. Symptoms of negative stress were found in 27.6% of the mothers in a moderate form and in 28.6% in a severe form.

### Prevalence of child behavior problems

Internalizing problems were observed in 42.0% of female children and 46.4% of male children, with an overall proportion of 44.0%. Externalizing behavior problems were reported in 54.9% of females and 48.5% of males, resulting in a total proportion of 51.9%. On the CBCL total score scale, 59.1% of female children and 59.5% of male children exhibited symptoms within the clinical range, with an overall proportion of 59.3% (Table 1).

### The relationship between maternal mental health and child behavior

The logistic regression analyses demonstrate significant associations between maternal mental health during pregnancy and child behavior at age seven (Table 2).

**Table 2:** Crude and adjusted logistic regression analyses (n = 508)

			Crude OR (95% CI)			Adjusted OR (95% CI)		
			Child behavior problems					
			Internal problems	External problems	Total problems	Internal problems	External problems	Total problems
Maternal mental health during pregnancy	Depression	normal	Ref.	Ref.	Ref.	Ref.	Ref.	Ref.
		mild	1.02 (0.59–1.79)	1.30 (0.76–2.25)	1.16 (0.68–1.98)	0.96 (0.71–1.29)	1.08 (0.80–1.47)	1.03 (0.78–1.37)
		moderate	**2.24 (1.39–3.62)**	1.48 (0.91–2.39)	**2.22 (1.35–3.66)**	**1.49 (1.14–1.95)**	1.15 (0.87–1.53)	**1.38 (1.05–1.80)**
		(extremely) severe	**1.92 (1.08–3.44)**	**2.53 (1.33–4.84)**	**3.17 (1.64–6.14)**	**1.39 (1.01–1.93)**	**1.44 (1.00–2.07.00.07)**	**1.58 (1.12–2.24)**
	Anxiety	normal	Ref.	Ref.	Ref.	Ref.	Ref.	Ref.
		mild	1.42 (0.81–2.51)	0.71 (0.41–1.23)	1.00 (0.57–1.73)	1.30 (0.95–1.77)	0.88 (0.64–1.20)	1.09 (0.79–1.51)
		moderate	**1.82 (1.15–2.89)**	**1.64 (1.02–2.63)**	**1.99 (1.24–3.21)**	**1.32 (1.02–1.69)**	1.21 (0.93–1.58)	**1.40 (1.06–1.84)**
		(extremely) severe	**2.15 (1.27–3.66)**	**2.23 (1.25–3.98)**	**2.58 (1.45–4.59)**	**1.36 (1.02–1.83)**	1.33 (0.96–1.84)	**1.43 (1.03–2.00.03.00)**
	Stress	normal	Ref.	Ref.	Ref.	Ref.	Ref.	Ref.
		mild	1.38 (0.77–2.49)	1.00 (0.56–1.77)	1.37 (0.77–2.44)	1.23 (0.89–1.69)	1.10 (0.80–1.53)	1.30 (0.93–1.82)
		moderate	1.35 (0.84–2.19)	1.30 (0.81–2.09)	**1.64 (1.03–2.62)**	1.21 (0.93–1.57)	1.12 (0.86–1.46)	1.26 (0.96–1.65)
		(extremely) severe	**1.69 (1.05–2.72)**	1.23 (0.77–1.96)	**2.22 (1.39–3.57)**	1.22 (0.94–1.59)	1.08 (0.82–1.41)	**1.41 (1.07–1.86)**
	* adjusted for maternal education, child´s sex, presence of siblings, maternal partnership status at baseline, maternal experiences of abuse, smoking during pregnancy, alcohol consumption during pregnancy, teenage pregnancy, preeclampsia, and low birth weight							

After accounting for these factors, moderate to severe maternal symptoms of depression and anxiety remained significantly associated with both internalizing and externalizing problems in children. Moderate maternal depression was linked to a higher risk of internalizing problems (OR = 1.49, 95% CI = 1.14–1.95) and total behavior problems (OR = 1.38, 95% CI = 1.05–1.80). Severe symptoms of depression increased the risk of internalizing (OR = 1.39, 95% CI = 1.01–1.93) and externalizing problems (OR = 1.44, 95% CI = 1.00–2.07.00.07). Severe maternal anxiety was significantly associated with internalizing (OR = 1.36, 95% CI = 1.02–1.83) and total behavior problems (OR = 1.43, 95% CI = 1.03–2.00.03.00). Additionally, severe maternal stress in pregnancy showed a significant association with total child behavior problems (OR = 1.41, 95% CI = 1.07–1.86).

### The role of child’s sex

Stratified logistic regression analyses adjusted for influencing covariates were conducted based on the child’s sex, where differences between females and males emerged (Table 3).

**Table 3:** Adjusted logistic regression stratified by child’s sex* (*n* = 506)

			Child behavior problems					
			Internal problems		External problems		Total problems	
			Females	Males	Females	Males	Females	Males
Maternal mental health during pregnancy	Depression	normal	Ref.	Ref.	Ref.	Ref.	Ref.	Ref.
		mild	1.14 (0.75–1.72)	0.85 (0.56–1.29)	1.21 (0.79–1.84)	1.10 (0.73–1.66)	1.09 (0.72–1.66)	0.98 (0.65–1.46)
		moderate	**1.62 (1.12–2.36)**	1.36 (0.94–1.97)	1.02 (0.70–1.47)	1.22 (0.82–1.81)	**1.54 (1.03–2.89)**	1.20 (0.82–1.77)
		(extremely) severe	**1.68 (1.09–2.60)**	1.13 (0.70–1.84)	1.54 (0.95–2.47)	1.34 (0.79–2.28)	**2.20 (1.28–3.77)**	1.18 (0.71–1.96)
	Anxiety	normal	Ref.	Ref.	Ref.	Ref.	Ref.	Ref.
		mild	1.12 (0.75–1.68)	1.58 (0.99–2.53)	0.85 (0.59–1.25)	0.81 (0.51–1.30)	0.86 (0.58–1.26)	1.33 (0.82–2.17)
		moderate	**2.13 (1.47–3.08)**	0.93 (0.65–1.32)	**1.60 (1.09–2.34)**	1.03 (0.72–1.47)	**2.37 (1.54–3.64)**	0.98 (0.69–1.38)
		(extremely) severe	**1.84 (1.21–2.78)**	1.12 (0.75–1.66)	1.26 (0.83–1.92)	1.48 (0.93–2.34)	**1.63 (1.04–2.56)**	1.26 (0.82–1.93)
	Stress	normal	Ref.	Ref.	Ref.	Ref.	Ref.	Ref.
		mild	1.17 (0.77–1.78)	1.38 (0.86–2.23)	1.08 (0.72–1.60)	1.02 (0.62–1.69)	0.99 (0.66–1.48)	**1.70 (1.01–2.86)**
		moderate	**1.48 (1.03–2.11)**	0.97 (0.68–1.38)	1.37 (0.96–1.95)	0.95 (0.66–1.37)	1.35 (0.94–1.93)	1.17 (0.82–1.67)
		(extremely) severe	**1.62 (1.13–2.34)**	1.03 (0.71–1.48)	1.14 (0.81–1.62)	0.93 (0.63–1.37)	**1.48 (1.03–2.13)**	1.30 (0.89–1.89)
	* adjusted for maternal education, presence of siblings, maternal partnership status at baseline, maternal experiences of abuse, smoking during pregnancy, alcohol consumption during pregnancy, teenage pregnancy, preeclampsia, and low birth weight							

Female children were particularly affected, exhibiting increased odds of internalizing and externalizing problems when exposed to maternal depressive, anxious, and stress symptoms during pregnancy. Moderate maternal anxiety symptoms more than doubled the odds of internalizing symptoms in females (OR = 2.13, 95% CI = 1.47–3.08), while severe anxiety also showed a strong association (OR = 1.84, 95% CI = 1.21–2.78). Similarly, moderate (OR = 1.48, 95% CI = 1.03–2.11) and severe maternal stress symptoms (OR = 1.62, 95% CI = 1.13–2.34) increased the odds of internalizing problems in female children. Moderate maternal depressive symptoms further heightened the odds (OR = 1.62, 95% CI = 1.12–2.36), as well as severe maternal depression (OR = 1.68, 95% CI = 1.09–2.60). The externalizing behaviors among females, such as rule-breaking and aggression, were significantly increased by moderate maternal anxiety symptoms during pregnancy (OR = 1.60, 95% CI = 1.09–2.34). When considering the total behavioral difficulties among females, severe maternal depressive symptoms were associated with increased odds (OR = 2.20, 95% CI = 1.28–3.77). Similarly, moderate maternal anxiety symptoms increased the probability of total problems in females (OR = 2.37, 95% CI = 1.54–3.64), while severe anxiety also showed a significant association (OR = 1.63, 95% CI = 1.04–2.56). Additionally, severe maternal stress symptoms (OR = 1.48, 95% CI = 1.03–2.13) increased the odds of total behavioral problems in female children.

In contrast to those findings, male children in the study were affected only by mild maternal stress symptoms, as reflected in the total problem score (OR = 1.70, 95% CI = 1.01–2.86). No significant associations were found between depression and anxiety in the mother and internal and external problems in male children.

Complete case analyses are shown in the appendix (Suppl. Tables 1–2). Overall, the results were similar to those from the imputed model, but with less precision due to the smaller sample size.

## Discussion

This study presents longitudinal findings on the association between prenatal maternal mental health and behavior problems in children at age seven, while accounting for several established risk factors. Results indicate that maternal symptoms of depression, anxiety, and stress during pregnancy are significantly associated with internalizing, externalizing, and total behavior problems in children from socioeconomically disadvantaged backgrounds. Sex-stratified analysis further revealed notable differences, with stronger associations observed in female children.

These findings align with existing research that links maternal mental health during pregnancy to behavioral problems in early childhood [36, 42].

The underlying mechanisms are likely rooted in a complex interplay of biological, psychological, and social factors. Previous studies have shown that newborns whose mothers experienced elevated prenatal anxiety or stress—often reflected in increased cortisol levels—were more restless, cried more, and had more difficult temperaments [9, 13] Van den Bergh [41, 46]. These early behavioral difficulties may persist over time, as elevated prenatal stress has also been linked to childhood anxiety, ADHD symptoms, and both internalizing and externalizing problems [10, 17, 42]. Importantly, the timing of exposure appears to matter. Maternal negative emotions between the 12th and 22nd weeks of pregnancy have been particularly associated with later behavioral problems, while effects were less evident in later gestational stages [42].

Genetic factors may also contribute to these associations. Familial clustering of depression and anxiety disorders is largely explained by genetic influences, as demonstrated by meta-analyses [20, 40]. This may partially explain why children of mothers experiencing psychological distress are at higher risk for behavioral problems.

### Sex differences

A key finding of this study is the sex-specific variation in the relationship between maternal mental health and child behavioral outcomes, consistent with prior studies [4, 7, 11, 11, 12, 21], traditional gender roles seem to influence both the expression and reporting of behavioral problems [35, 39].

These roles may also shape maternal parenting behavior and attachment patterns toward daughters and sons, thereby influencing how mental health risks are transmitted across generations [6, 7, 19, 27]. This includes the interaction between mother and child in infancy which differs by gender as well [31]. Consistent with previous research [23], our results indicated a dose-response relationship between maternal mental health issues and behavioral problems. However, there was a notable exception among male children, where mild but not severe stress symptoms were associated with behavioral problems. As there is no plausible explanation for this pattern, further research is required to confirm this finding.

It is also important to note that CBCL T-scores are standardized using sex-specific norms. Lower raw scores in females can yield elevated T-scores, meaning males and females with similar behaviors may receive different classifications. As a result, T-scores are not directly comparable between sexes, which should be considered when interpreting these findings.

### Strengths and limitations

This study contributes to a relatively underexplored area by examining the association between maternal mental health and later behavioral problems in socioeconomically disadvantaged populations. The consistency between unadjusted and adjusted logistic regression models enhances confidence in the robustness of the results. Additional strengths include the use of well-validated instruments for assessing maternal mental health and child behavioral outcomes, a relatively low attrition rate over the seven-year follow-up period in a high-risk sample, and multiple imputation techniques to address missing data. Notably, the results with the imputed dataset were consistent with the complete case analyses, increasing confidence in the reliability of the findings.

However, some limitations should be acknowledged. Both the DASS-21 and CBCL rely on maternal self-report, which may introduce reporting bias. Prior research suggests internalizing problems may be underreported, while externalizing behaviors may be overestimated [24]. Conversely, mothers experiencing severe psychological distress may overreport internalizing problems [33, 43]. Incorporating additional informants, such as teachers, could provide a more comprehensive assessment. As no data on contact with the father or paternal mental health were available, these potentially influential factors could not be considered. This may have limited our ability to detect sex-specific pathways of risk transmission. Due to the specific distribution of risk factors among this study population (for example, all participants were socioeconomically deprived at baseline), our findings require confirmation in the general population. Finally, unmeasured confounding factors, such as genetic influences, cannot be fully excluded. Additionally, as this is an observational study, conclusions about causality should be drawn with caution.

## Conclusions

This study found that moderate to severe maternal depression, anxiety, or stress during pregnancy significantly increased the risk of behavioral problems in children at age seven in a socially disadvantaged population, with stronger effects observed in female children. These findings emphasize the importance of early identification and intervention for maternal psychological distress during pregnancy, particularly in socioeconomically disadvantaged populations, to support healthy child development. Future research should continue to explore sex-specific pathways and mechanisms to better understand and ultimately reduce the intergenerational transmission of mental health risks.

## Supplementary information

Below is the link to the electronic supplementary material.

ESM 1 (DOCX 22.5 KB)

## Data Availability

The data that support the findings of this study are not openly available due to reasons of sensitivity and are available from the corresponding author upon reasonable request.

## Abbreviations

(CBCL 61-8R): Child Behavior Checklist
(DASS-21): Depression Anxiety-Stress Scale
