# Supplementary material for: Sex-specific associations between prenatal maternal mental health and child behavior problems at age 7: A (multi-center) longitudinal study of socially disadvantaged mother-child dyads
Source: Eur Child Adolesc Psychiatry. 2026 Feb 10;35(5):1641–9. doi: 10.1007/s00787-026-02974-z (PMC13272258; doi:10.1007/s00787-026-02974-z)
Supplement: Supplementary file 1 — (DOCX 22.5 KB) [file 787_2026_2974_MOESM1_ESM.docx]

# Appendix

Supp. Table 1. Crude and adjusted* logistic regression analyses (complete case analyses)

|  |  |  | **Crude OR (95% CI)** | | | **Adjusted OR* (95% CI)** | | |
| --- | --- | --- | --- | --- | --- | --- | --- | --- |
|  |  |  | Child behavior problems | | | | | |
|  |  |  | Internal problems | External problems | Total problems | Internal problems | External problems | Total problems |
| Maternal mental health during pregnancy | Depression |  | n = 393 | n = 475 | n = 364 | n = 228 | n = 270 | n = 209 |
|  |  | normal | Ref. | Ref. | Ref. | Ref. | Ref. | Ref. |
|  |  | mild | 1.04 (0.56-1.93) | 1.23 (0.71-2.15) | 1.17 (0.63-2.19) | 0.81 (0.35-1.84) | 1.62 (0.70-3.76) | 1.22 (0.49-3.07) |
|  |  | moderate | **1.96 (1.14-3.35)** | 1.28 (0.79-2.07) | **2.98 (1.60-5.57)** | 1.49 (0.71-2.14) | 1.12 (0.55-2.27) | 2.14 (0.88-5.24) |
|  |  | (extremely) severe | **2.59 (1.35-4.94)** | **2.83 (1.49-5.38)** | **4.49 (1.90-10.61)** | 1.64 (0.61-4.42) | 1.50 (0.58-3.93) | 2.67 (0.76-9.32) |
|  | Anxiety |  | n = 392 | n = 473 | n = 363 | n = 227 | n = 269 | n = 208 |
|  |  | normal | Ref. | Ref. | Ref. | Ref. | Ref. | Ref. |
|  |  | mild | 1.59 (0.84-3.01) | 0.81 (0.45-1.46) | 1.35 (0.69-2.67) | 1.65 (0.69-3.96) | 0.91 (0.40-2.08) | 2.42 (0.86-6.82) |
|  |  | moderate | **1.78 (1.07-2.97)** | **1.82 (1.14-2.92)** | 1.60 (0.92-2.78) | 1.56 (0.78-3.12) | 1.34 (0.69-2.60) | 1.47 (0.66-3.25) |
|  |  | (extremely) severe | **1.90 (1.06-3.43)** | **2.28 (1.30-3.99)** | **3.30 (1.64-6.64)** | 1.44 (0.63-3.29) | 1.91 (0.84-4.35) | **3.08 (1.08-8.84)** |
|  | Stress |  | n = 392 | n = 474 | n = 363 | n = 227 | n = 269 | n = 208 |
|  |  | normal | Ref. | Ref. | Ref. | Ref. | Ref. | Ref. |
|  |  | mild | 1.47 (0.78-2.79) | 1.22 (0.68-2.19) | 1.09 (0.57-2.09) | 1.39 (0.59-3.26) | 1.20 (0.53-2.73) | 1.01 (0.40-2.59) |
|  |  | moderate | 1.52 (0.89-2.61) | 1.42 (0.88-2.28) | **1.90 (1.09-3.32)** | 1.44 (0.71-2.95) | 1.29 (0.66-2.52) | 1.39 (0.62-3.11) |
|  |  | (extremely) severe | **1.95 (1.15-3.31)** | 1.48 (0.92-2.38) | **3.62 (2.02-6.52)** | 1.47 (0.70-3.07) | 1.38 (0.70-2.74) | **4.45 (1.77-11.17)** |
|  | * adjusted for maternal education, child´s sex, presence of siblings, maternal partnership status at baseline, maternal experiences of abuse, smoking during pregnancy, alcohol consumption during pregnancy, teenage pregnancy, pre-eclampsia, and low birth weight | | | | | | | |

Supp. Table 2: Adjusted* odds ratio, stratified by child’s sex (complete case analyses)

|  |  |  | Child behavior problems | | | | | |
| --- | --- | --- | --- | --- | --- | --- | --- | --- |
|  |  |  | Internal problems | | External problems | | Total problems | |
|  |  |  | Females | Males | Females | Males | Females | Males |
| Maternal mental health during pregnancy | Depression |  | n = 132 | n = 96  Ref.  0.31 (0.08-1.22)  1.83 (0.54-6.24)  1.05 (0.20-5.43) | n = 149  Ref.  2.85 (0.79-10.24)  0.99 (0.36-2.76)  0.76 (0.19-2.98) | n = 121  Ref.  1.22 (0.34-4.35)  1.22 (0.40-3.73)  1.63 (0.32-8.27) | n = 121  Ref.  1.99 (0.52-7.54)  2.45 (0.66-9.07)  3.28 (0.51-21.13) | n = 88  Ref.  0.99 (0.23-4.35)  2.51 (0.52-12.09)  0.86 (0.09-8.11) |
|  |  | normal | Ref. |  |  |  |  |  |
|  |  | mild | 1.68 (0.53-5.30) |  |  |  |  |  |
|  |  | moderate | 1.53 (0.54-4.32) |  |  |  |  |  |
|  |  | (extremely) severe | 2.84 (0.70-11.60) |  |  |  |  |  |
|  | Anxiety |  | n = 131  Ref.  1.68 (0.52-5.40)  **3.12 (1.18-8.27)**  2.93 (0.84-10.20) | n = 96  Ref.  1.19 (0.24-5.81)  0.75 (0.25-2.28)  0.82 (0.24-2.74) | n = 148  Ref.  0.75 (0.25-2.26)  2.10 (0.78-5.66)  1.10 (0.32-3.79) | n = 121  Ref.  1.01 (0.24-4.17)  0.82 (0.28-2.35)  2.12 (0.62-7.26) | n = 120  Ref.  1.69 (0.44-6.45)  **4.89 (1.37-17.49)**  1.64 (0.35-7.63) | n = 88  Ref.  4.31 (0.48-38.85)  0.54 (0.14-2.18)  5.64 (0.90-35.21) |
|  |  | normal |  |  |  |  |  |  |
|  |  | mild |  |  |  |  |  |  |
|  |  | moderate |  |  |  |  |  |  |
|  |  | (extremely) severe |  |  |  |  |  |  |
|  | Stress |  | n = 131  Ref.  1.91 (0.60-6.07)  **3.14 (1.13-8.72)**  **3.30 (1.05-10.38)** | n = 96  Ref.  2.17 (0.45-10.47)  0.61 (0.20-1.86)  0.80 (0.25-2.61) | n = 148  Ref.  1.20 (0.43-3.39)  2.17 (0.81-5.79)  1.32 (0.47-3.74) | n = 121  Ref.  1.15 (0.23-5.82)  0.84 (0.30-2.35)  0.91 (0.30-2.75) | n = 120  Ref.  1.08 (0.33-3.56)  1.50 (0.50-4.52)  **5.81 (1.28-26.34)** | n = 88  Ref.  0.50 (0.07-3.77)  1.52 (0.39-5.89)  1.90 (0.43-8.38) |
|  |  | normal |  |  |  |  |  |  |
|  |  | mild |  |  |  |  |  |  |
|  |  | moderate |  |  |  |  |  |  |
|  |  | (extremely) severe |  |  |  |  |  |  |
|  | * adjusted for maternal education, presence of siblings, maternal partnership status at baseline, maternal experiences of abuse, smoking during pregnancy, alcohol consumption during pregnancy, teenage pregnancy, pre-eclampsia, and low birth weight | | | | | | | |
